# Association of Neighborhood Conditions and Resources for Children With Life Expectancy at Birth in the US

**DOI:** 10.1001/jamanetworkopen.2022.35912

**Published:** 2022-10-14

**Authors:** Kristen H. Shanahan, S. V. Subramanian, Kendall J. Burdick, Michael C. Monuteaux, Lois K. Lee, Eric W. Fleegler

**Affiliations:** 1 Department of Emergency Medicine, Massachusetts General Hospital, Boston; 2 Division of Emergency Medicine, Boston Children’s Hospital, Boston, Massachusetts; 3 Department of Pediatrics, Harvard Medical School, Boston, Massachusetts; 4 Department of Social and Behavioral Sciences, Harvard T.H. Chan School of Public Health, Boston, Massachusetts; 5 University of Massachusetts Medical School, Worcester

## Abstract

**Importance:**

To address inequities in life expectancy, we must understand the associations of modifiable socioeconomic and structural factors with life expectancy. However, the association of limited neighborhood resources and deleterious physical conditions with life expectancy is not well understood.

**Objective:**

To evaluate the association of community social and economic conditions and resources for children with life expectancy at birth.

**Design, Setting, and Participants:**

This cross-sectional study examined neighborhood child opportunity and life expectancy using data from residents of 65 662 US Census tracts in 2015. The analysis was conducted from July 6 to October 1, 2021.

**Exposures:**

Neighborhood conditions and resources for children in 2015.

**Main Outcomes and Measures:**

The primary outcome was life expectancy at birth at the Census tract level based on data from the US Small-Area Life Expectancy Estimates Project (January 1, 2010, to December 31, 2015). Neighborhood conditions and resources for children were quantified by Census tract Child Opportunity Index (COI) 2.0 scores for 2015. This index captures community conditions associated with children’s health and long-term outcomes categorized into 5 levels, from very low to very high opportunity. It includes 29 indicators in 3 domains: education, health and environment, and social and economic factors. Mixed-effects and simple linear regression models were used to estimate the associations between standardized COI scores (composite and domain-specific) and life expectancy.

**Results:**

The study included residents from 65 662 of 73 057 US Census tracts (89.9%). Life expectancy at birth across Census tracts ranged from 56.3 years to 93.6 years (mean [SD], 78.2 [4.0] years). Life expectancy in Census tracts with very low COI scores was lower than life expectancy in Census tracts with very high COI scores (−7.06 years [95% CI, −7.13 to −6.99 years]). Stepwise associations were observed between COI scores and life expectancy. For each domain, life expectancy was shortest in Census tracts with very low compared with very high COI scores (education: β = −2.02 years [95% CI, −2.12 to −1.92 years]); health and environment: β = −2.30 years [95% CI, −2.41 to −2.20 years]; social and economic: β = −4.16 years [95% CI, −4.26 to −4.06 years]). The models accounted for 41% to 54% of variability in life expectancy at birth (*R*^2^ = 0.41-0.54).

**Conclusions and Relevance:**

In this study, neighborhood conditions and resources for children were significantly associated with life expectancy at birth, accounting for substantial variability in life expectancy at the Census tract level. These findings suggest that community resources and conditions are important targets for antipoverty interventions and policies to improve life expectancy and address health inequities.

## Key Points

**Question:**

Are neighborhood conditions and resources for children associated with life expectancy at birth in the US?

**Findings:**

In this cross-sectional study of residents from 65 662 US Census tracts, neighborhood conditions and resources for children explained substantial variability in life expectancy at birth. Neighborhood conditions and resources for children had a stepwise association with life expectancy at birth, with the lowest life expectancy in the communities with the lowest neighborhood conditions and resources for children.

**Meaning:**

The study’s findings suggest that neighborhood conditions and resources for children are potentially important targets for health policy aimed at improving life expectancy for socially vulnerable populations in the US.

## Teaser

This cross-sectional study assesses the associations of neighborhood socioeconomic conditions and resources for children with life expectancy at birth in the US.

## Introduction

Health inequities in socially vulnerable populations are pervasive in the US. The importance of this issue is illustrated by inequities in life expectancy. In the US, one of the primary factors associated with life expectancy is location of residence.^1,2,3,4,5^ Life expectancy across US Census tracts ranges more than 40 years, from 56 years to 97 years.^1,2^

Neighborhood poverty is associated with adverse health outcomes and higher mortality.^6,7,8,9,10,11^ Limited neighborhood resources and deleterious physical conditions are a manifestation of community poverty. The terms *neighborhood opportunity* and *geography of opportunity* describe community-level conditions that have implications for child development, long-term health, and socioeconomic outcomes.^12,13,14,15,16^ These community-level conditions include educational, health, environmental, and socioeconomic factors.^12,13,14,15,16^ The social and environmental conditions in which people live likely account for part of the association between neighborhood poverty and health.^17^ Neighborhood opportunity for children has been associated with health outcomes in children, including hospitalizations, acute care visits, infant health, mental health, and cardiometabolic risk factors.^18,19,20,21,22,23,24,25,26^

Interventions to improve life expectancy in socially vulnerable populations are challenging but possible. To develop interventions, we must first better understand the associations between life expectancy and community-level factors. Specific policies and interventions can then be implemented to target specific factors, such as employment, education, and the physical environment, at the neighborhood level.^27^ Neighborhood opportunity for children offers this actionable potential; by investing in communities to improve neighborhood resources and conditions for children, we may alter health outcomes across the lifespan. However, gaps in knowledge exist regarding the impact of neighborhood opportunity for children on life expectancy.

The objectives of this study were to (1) evaluate the association between neighborhood opportunity for children and life expectancy at birth in the US at the Census tract level and (2) compare the associations of different domains of neighborhood opportunity with life expectancy at birth. Our hypothesis was that life expectancy at birth would be lower in communities with low neighborhood opportunity for children. We expected that use of an index inclusive of multiple facets of neighborhood opportunity for children may offer insight into mechanisms by which neighborhood opportunity has implications for life expectancy. A better understanding of this association could potentially identify neighborhood opportunity for children as an actionable target for health policy, community intervention, and research to improve life expectancy in vulnerable populations in the US.

## Methods

We conducted a cross-sectional study of neighborhood child opportunity and life expectancy in the US using 2 sources of data: the Child Opportunity Index (COI) 2.0^16^ for 2015 and the US Small-Area Life Expectancy Estimates Project,^1^ which used data from January 1, 2010, to December 31, 2015. Data analysis was conducted from July 6 to October 1, 2021. The study was considered exempt from review and the requirement for informed consent by the institutional review board of Boston Children’s Hospital due to the use of publicly available deidentified data. This study followed the Strengthening the Reporting of Observational Studies in Epidemiology (STROBE) guideline for cross-sectional studies.

### Neighborhood Child Opportunity

Neighborhood child opportunity was measured using the COI 2.0.^16^ This index captures community conditions and resources associated with children’s health and long-term outcomes. It includes 29 weighted indicators of neighborhood conditions and classifies the indicators in 3 domains: education, health and environment, and social and economic factors (eTable 1 in the Supplement).

Raw COI scores and standardized *z* scores were generated for 72 195 of 73 057 US Census tracts (98.8%) for 2015. The creators of the COI excluded Census tracts that were fully covered by water or had missing data for greater than 50% of the indicators in any of the 3 domains.^28^ Each Census tract has an overall COI score, which represents performance on 29 indicators of neighborhood conditions and resources. Each Census tract also has scores for each of the 3 domains (education, health and environment, and social and economic factors) as well as individual scores for each of the 29 indicators. Higher index scores represent more favorable neighborhood conditions, such as more green spaces, higher median household income, and better access to early childhood education.

After scores were calculated, Census tracts were divided into 5 ordinal groups representing quintiles of nationally normed COI scores ranging from 0 to 100. The 5 groups of Census tracts were identified as very low opportunity (score range, 0 to <20), low opportunity (score range, 20 to <40), moderate opportunity (score range, 40 to <60), high opportunity (score range, 60 to <80), and very high opportunity (score range, 80 to 100). Grouping by quintiles was performed for the overall COI scores and the 3 domains. The groups were generated by the creators of the index, with published methods.^15,16,28^

### Life Expectancy

We obtained life expectancy data from the US Small-Area Life Expectancy Estimates Project of the Centers for Disease Control and Prevention.^1,29^ The project generated life expectancy estimates for US Census tracts using cross-sectional mortality data from 2010 to 2015. Each Census tract has a single projected life expectancy at birth and age 1 year for the entire 6-year period. Data were not available for Maine, Wisconsin, and any Census tracts with a 6-year total population of fewer than 5000 people.

### Outcome and Exposures

The primary outcome was life expectancy at birth in the US at the Census tract level. A sensitivity analysis for life expectancy at age 1 year was also performed. The primary exposure was the COI overall score for the Census tract in 2015. Secondary exposures included COI domain-specific and individual component scores.

### Statistical Analysis

Descriptive statistics were used to characterize the COI scores and life expectancy at birth for the Census tracts using means and SDs. For the primary model, we used mixed-effects linear regression analysis to estimate the association of the overall COI score with life expectancy at birth at the Census tract level. Individual-level data on age, sex, race, and ethnicity were not available from the US Small-Area Life Expectancy Estimates Project. The model included a fixed effect for the COI, random intercepts at the state and county levels, and a random slope for the COI. We visually evaluated the homoscedasticity and normality of residuals by generating a scatterplot of the residuals vs the estimated values and examining a histogram of the residuals. The assumptions of the linear model were satisfied. The overall COI score was used in the model in the form of the 5 ordinal groups (very low, low, moderate, high, and very high opportunity), as previously described.^16,28^ These categories were modeled as a set of indicator variables, with the very high opportunity category set as the referent.

For the secondary analyses, we aimed to understand whether the association between neighborhood child opportunity and life expectancy may be explained by specific elements of neighborhood conditions. First, we used mixed-effects linear regression analysis to estimate the association between the 3 domains of the COI and life expectancy at birth at the Census tract level. We used COI scores for the 3 domains: education, health and environment, and social and economic (eTable 1 in the Supplement). The domain scores were used in the form of the 5 ordinal groups (very low, low, moderate, high, and very high opportunity), as previously described.^16,28^

For the next set of secondary analysis, we used mixed-effects linear regression models to estimate the association between the 29 indicators that comprise the COI and life expectancy at birth. We used nationally normed raw scores for each indicator; no groupings were used in this model. We assessed for collinearity and excluded indicators with a variance inflation factor greater than 10.

We performed several sensitivity analyses. First, we estimated simple linear regression models to test the association between COI score and life expectancy at birth for Census tracts, with 1 model each for overall scores, domain scores, and indicator scores. The models for overall and domain scores used the 5 ordinal categories of opportunity. The model of the indicator scores used raw scores. Next, we performed sensitivity analyses using mixed-effects linear regression models to estimate the associations of the overall and domain-specific COI categories for the neighborhood with life expectancy at age 1 year, with the same parameters as the primary model. These analyses permitted us to evaluate whether exclusion of infant mortality substantially changed the association. Collinearity diagnostic assessments were performed. All mixed-effects linear regression models included random intercepts for state and county. Simple linear regression models used robust SEs clustered on county to account for correlation among Census tracts within the same county. We calculated β and *R*^2^ coefficients for the outcomes, with β coefficients reflecting the change in life expectancy per 1-step increase in category of COI score (very low, low, moderate, high, and very high opportunity). All tests were 2-tailed, and significance was set at α = .05. Statistical analyses were conducted using Stata SE software, version 15 (StataCorp LLC).

## Results

**Figure 1.**
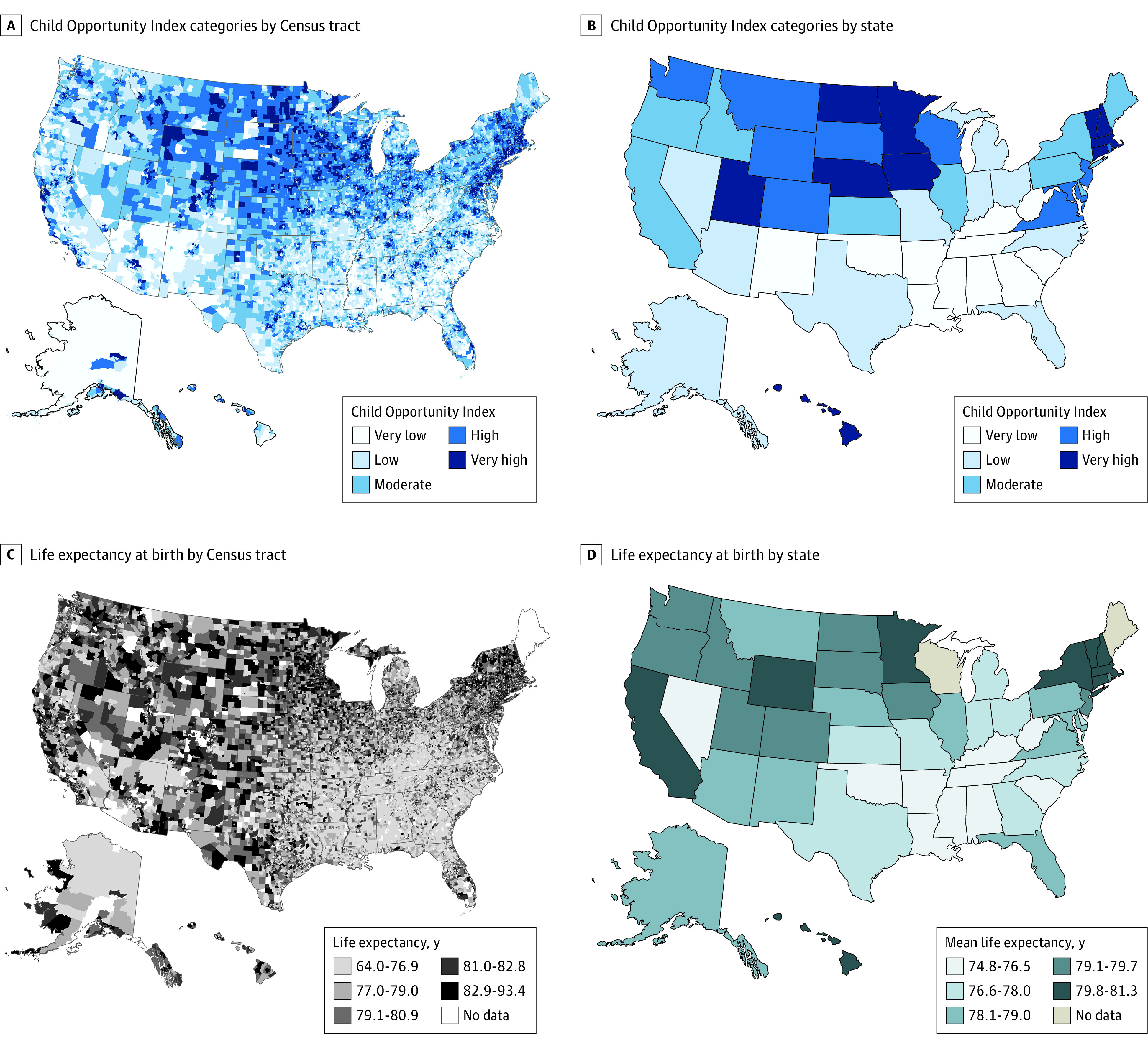
Maps of Child Opportunity Index Categories and Life Expectancy at Birth by US Census Tract and State Census tracts were categorized into 5 groups based on nationally normed Child Opportunity Index scores (range, 0-100), with very low opportunity indicating 0 to <20; low opportunity, 20 to <40; moderate opportunity, 40 to <60; high opportunity, 60 to <80; and very high opportunity, 80 to 100. Maps include all available data prior to exclusion of census tracts for missing data in linked data set.

Among 73 057 US Census tracts, 4703 were excluded because they lacked life expectancy estimates from the Small-Area Life Expectancy Estimates Project, and 2692 were excluded because they had incomplete data from the COI. The remaining 65 662 Census tracts (89.9%) were included in the analysis (eFigure 1 in the Supplement). The range of the nationally normed COI composite scores was 0 to 100, with a mean (SD) of 49.0 (28.4) (Figure 1). The mean (SD) COI composite scores ranged from 10.1 (5.9) for the Census tracts with very low opportunity to 90.2 (5.7) for the Census tracts with very high opportunity (Table 1).

**Table 1.** COI Scores Across US Census Tracts With Very Low to Very High Neighborhood Opportunity for Children

COI category for Census tract^a^	COI score, mean (SD)				Life expectancy at birth, mean (SD), y
	Composite	Domain			
		Education	Health and environment	Social and economic	
Very low	10.1 (5.9)	18.9 (15.5)	18.6 (18.3)	10.7 (6.9)	74.4 (3.7)
Low	30.6 (5.8)	34.1 (18.1)	36.3 (21.5)	31.2 (8.5)	77.1 (3.2)
Moderate	50.5 (5.8)	48.4 (19.0)	51.1 (22.8)	50.9 (9.7)	78.5 (3.0)
High	70.4 (5.7)	64.8 (17.9)	65.1 (21.3)	70.6 (9.9)	80.1 (2.7)
Very high	90.2 (5.7)	85.4 (12.4)	77.9 (17.5)	89.0 (8.1)	81.9 (2.6)

Abbreviation: COI, Child Opportunity Index 2.0.

^a^ Census tracts were categorized into 5 groups based on nationally normed COI scores (range, 0-100), with very low opportunity indicating 0 to <20; low opportunity, 20 to <40; moderate opportunity, 40 to <60; high opportunity, 60 to <80; and very high opportunity, 80 to 100.

**Figure 2.**
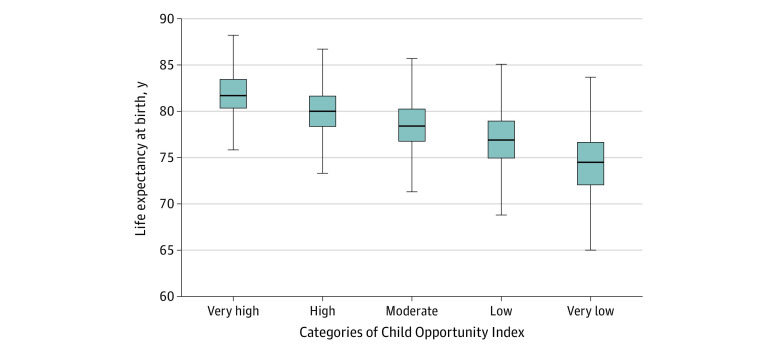
Life Expectancy at Birth by Child Opportunity Index Category Census tracts were categorized into 5 groups based on nationally normed Child Opportunity Index scores (range, 0-100), with very low opportunity indicating 0 to <20; low opportunity, 20 to <40; moderate opportunity, 40 to <60; high opportunity, 60 to <80; and very high opportunity, 80 to 100. Outside values were excluded. Whiskers represent 95% CIs.

The mean (SD) life expectancy at birth for all Census tracts was 78.2 (4.0) years. The difference among Census tracts with the lowest vs highest life expectancies was 37.3 years (range, 56.3-93.6 years) (Figure 1). The mean (SD) life expectancy at birth was 74.4 (3.7) years for Census tracts with very low opportunity and 81.9 (2.6) years for Census tracts with very high opportunity (Figure 2).

### Overall COI Category and Life Expectancy at Birth

Life expectancy in Census tracts with very low opportunity for children was lower than life expectancy in Census tracts with very high opportunity for children (−7.06 years [95% CI −7.13 to −6.99 years]) in the mixed-effects model (Table 2). We observed a stepwise association between overall COI category and life expectancy at birth, with significant differences between every level relative to the very high opportunity category (low opportunity: β = −4.50 years [95% CI, −4.58 to −4.43 years]; moderate opportunity: β = −3.02 years [95% CI, −3.09 to −2.95 years]; high opportunity: β = −1.60 years [95% CI, −1.67 to −1.53 years]). In the simple linear regression model, the overall COI category had a strong correlation with life expectancy, accounting for 41% of the variability in life expectancy at birth (*R*^2^ = 0.41) (eTable 2 in the Supplement).

**Table 2.** Mixed-Effects Linear Regression Models for COI and Life Expectancy at Birth

COI category for Census tract^a^	Change in life expectancy at birth, β (95% CI), y			
	Model for COI composite score	Model for COI domain-specific scores		
		Education	Health and environment	Social and economic
Very low	−7.06 (−7.13 to −6.99)	−2.02 (−2.12 to −1.92)	−2.30 (−2.41 to −2.20)	−4.16 (−4.26 to −4.06)
Low	−4.50 (−4.58 to −4.43)	−1.48 (−1.57 to −1.40)	−1.38 (−1.47 to −1.29)	−2.42 (−2.51 to −2.33)
Moderate	−3.02 (−3.09 to −2.95)	−1.16 (−1.24 to −1.08)	−0.83 (−0.92 to −0.75)	−1.59 (−1.67 to −1.51)
High	−1.60 (−1.67 to −1.53)	−0.72 (−0.80 − 0.65)	−0.43 (−0.50 to −0.36)	−0.87 (−0.94 to −0.80)
Very high	1 [Reference]	1 [Reference]	1[Reference]	1 [Reference]

Abbreviation: COI, Child Opportunity Index 2.0.

^a^ Census tracts were categorized into 5 groups based on nationally normed COI scores (range, 0-100), with very low opportunity indicating 0 to <20; low opportunity, 20 to <40; moderate opportunity, 40 to <60; high opportunity, 60 to <80; and very high opportunity, 80 to 100.

### Domains and Indicators of the COI and Life Expectancy at Birth

Life expectancy in Census tracts with very low opportunity for children was shorter than life expectancy in Census tracts with very high opportunity for children in each COI domain (education: β = −2.02 years [95% CI, −2.12 to −1.92 years]; health and environment: β = −2.30 years [95% CI, −2.41 to −2.20 years], social and economic: β = −4.16 years [95% CI, −4.26 to −4.06 years]) (Table 2). We similarly observed a stepwise association between lower COI scores and decreasing life expectancy at birth in all of the domains. In the simple linear regression model, the domain-specific categories of the COI accounted for 44% of the variability in life expectancy at birth (*R*^2^ = 0.44) (eTable 2 in the Supplement).

**Figure 3.**
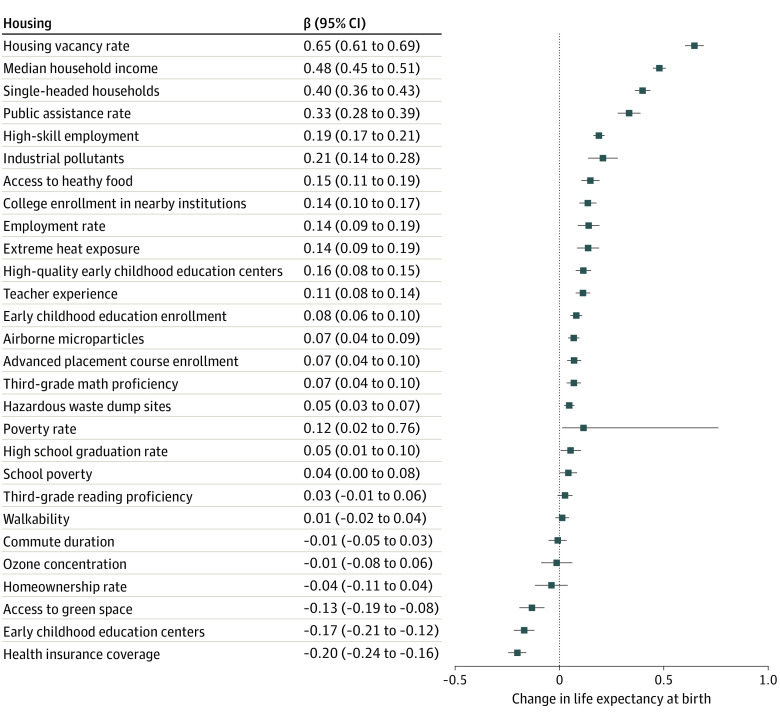
Change in Life Expectancy at Birth by Components of Child Opportunity Index Census tracts were categorized into 5 groups based on nationally normed Child Opportunity Index scores (range, 0-100), with very low opportunity indicating 0 to <20; low opportunity, 20 to <40; moderate opportunity, 40 to <60; high opportunity, 60 to <80; and very high opportunity, 80 to 100. Change in life expectancy was measured per 1-step increase in category of Child Opportunity Index.

Adult educational attainment was removed from the model due to collinearity with high-skill employment. Thus, the model of the individual components of the COI and life expectancy at birth included 28 indicators. The indicators associated with the greatest increases in life expectancy (β coefficient for years per 1-step increase in category of COI, ranging from very low to very high opportunity) were lower public assistance rate (β = 0.69; 95% CI, 0.64-0.73), lower housing vacancy rate (β = 0.48; 95% CI, 0.45-0.51), fewer single-headed households (β = 0.40; 95% CI, 0.36-0.43), and higher median household income (β = 0.33; 95% CI, 0.28-0.39) (Figure 3). The simple linear model accounted for 54% of the variability in life expectancy at birth (*R*^2^ = 0.54) (eFigure 2 in the Supplement).

### COI and Life Expectancy at Age 1 Year

In the analyses of COI scores and life expectancy at age 1 year, the mixed-effects regression model results were consistent with those of the primary model (eTable 3 in the Supplement). Life expectancy in Census tracts with very low opportunity for children was shorter than life expectancy in Census tracts with very high opportunity for children both overall (β = −6.77 years; 95% CI −6.84 to −6.70 years) and in each COI domain (education: β = −1.93 years [95% CI, −2.02 to −1.83 years]; health and environment: β = −2.20 years [95% CI, −2.31 to −2.10 years], social and economic: β = −4.00 years [95% CI, −4.10 to −3.90 years]).

## Discussion

In this national cross-sectional study of the association between neighborhood opportunity for children (which included measures of community resources and conditions) and life expectancy at birth, lower opportunity was significantly associated with a stepwise decrease in life expectancy. In our models, neighborhood opportunity for children, as measured by the COI, accounted for 41% to 54% of the variability in life expectancy at birth across US Census tracts. Among the domains, the social and economic scores were associated with the greatest differences in life expectancy. The focus on neighborhood resources and conditions for children may translate to actionable results by highlighting specific targets for health and social policy.

We propose several potential explanations for the association between neighborhood opportunity for children and life expectancy. First, community resources and conditions for children may have implications for children’s health, which is the foundation for health across the lifespan. Better access to high-quality education may lead to stronger employment potential and higher income, thereby affording families and children with better housing, nutrition, and health care access. Over time, these factors may improve health outcomes.^30,31^ Cleaner environmental conditions, which are associated with areas with higher COI levels, have implications for better health.^32,33,34^ Furthermore, rapid development of the brain and body during the prenatal and childhood stages may confer a distinct susceptibility to toxic exposures in areas with lower COI levels, with important long-term consequences for health.^35,36,37^

A second potential explanation for the findings is that community conditions and resources are associated with life expectancy across the lifespan, and neighborhood opportunity for children is simply a measure of neighborhood opportunity for all ages.^2,3,4^ Overlap was present between the COI indicators and community conditions for all ages. However, in this study, neighborhood conditions for children were associated with larger differences in life expectancy at birth compared with other socioeconomic and demographic factors for the overall population reported in a previous study.^2^ This finding suggests that life expectancy may potentially be adversely affected by community exposures during childhood, such as neighborhood poverty, access to healthy food, and toxic environmental exposures.

To our knowledge, this study is the first to specifically examine the association of neighborhood resources and conditions for children with life expectancy. The previously published literature on the COI has suggested an association between lower neighborhood opportunity for children and worse health outcomes among children.^18,19,20,21,22,23,24,25^ Our findings add to this body of evidence by suggesting that neighborhood opportunity for children may not only have implications for outcomes in children but may also be associated with life expectancy, a long-term health outcome. By focusing on childhood community exposures, we expanded on previous research that found an association between individual-level socioeconomic and demographic factors (including place of residence, race and ethnicity, and income) and life expectancy in the US.^3,4,38,39,40,41^ This study identified neighborhood conditions and resources in the domains of education, health and environment, and social and economic contexts for children as potential factors associated with life expectancy that may partially explain the established association between location of residence and life expectancy.^2,3,4,5^

This study’s findings have implications for health policy and research within the context of improving life expectancy for socially vulnerable populations. Although location of residence is significantly associated with life expectancy,^2,3,4,5^ it is not a risk factor easily changed at the population or individual level. Our findings suggest that community resources and conditions for children, which are potentially modifiable exposures, should be an important target for health policy and interventions to address inequities in life expectancy. In the domain of social and economic factors, policies aimed at providing better financial support for families, extending the child tax credit, expanding Medicaid, and supporting nutritional assistance for families, specifically among socially vulnerable populations, should be further explored. In the domain of health and environmental factors, the focus may include efforts to increase healthy environments through less pollution and more green spaces, reductions in environmental toxins, and expansion of access to better housing and healthy food. In the domain of educational factors, improving public education and expanding access to early childhood education may help to improve health outcomes. The data suggest that higher median income, lower public assistance, lower housing vacancy rates, and fewer single-headed households are most significantly associated with higher life expectancy. These findings highlight the importance of interventions that aim to reduce poverty and housing insecurity.

For the field of research, this study raises the question of whether neighborhood opportunity for children may partially explain other inequities in life expectancy, including variability by race and ethnicity. The data suggest the community poverty rate may underestimate associations in investigations of social and economic contributors to health outcomes when compared with more comprehensive indices of community conditions and resources, such as the COI. Future research on neighborhood opportunity for children and life expectancy should (1) examine whether neighborhood opportunity for children mediates the association of race and ethnicity with life expectancy, (2) explore how changes in specific elements of neighborhood opportunity for children may be associated with changes in health, and (3) quantify changes in life expectancy in response to policy and community interventions to improve neighborhood opportunity for children.

### Limitations

This study has limitations, including the use of ecological data, which are subject to bias and do not permit causal conclusions. However, the study used 2 robust national sources of data at the Census tract level, which allowed for granular investigation of community-level associations. Lack of life expectancy estimates by race and ethnicity at the Census tract level from the US Small-Area Life Expectancy Estimates Project precluded exploration of structural racism and the ways in which neighborhood opportunity for children may partially explain the association of race and ethnicity with life expectancy. Low neighborhood opportunity for children concentrates heavily among Black and Hispanic children in the US; 66% of Black children and 58% of Hispanic children live in communities with very low or low opportunity for children compared with 18% of White children and 27% of Asian and Pacific Islander children.^16^ Therefore, this lack of data on race and ethnicity is a notable limitation of this study and an important component for future investigations. Furthermore, life expectancy data are not available for Census tracts with 6-year populations of fewer than 5000 people, possibly excluding rural areas.

## Conclusions

In this cross-sectional study, neighborhood opportunity for children was significantly associated with life expectancy at birth in the US, both in aggregate and across all domains (education, health and environment, and social and economic factors). Specific interventions could be developed within these domains to improve health outcomes, including life expectancy. These findings suggest neighborhood opportunity for children may be an important target for health policy and research aimed at improving inequities in life expectancy across the US.

## Notes

Supplement. **eTable 1.** Indicators of the Child Opportunity Index **eTable 2.** Simple Linear Regression Analysis of Child Opportunity Index and Life Expectancy at Birth **eTable 3.** Mixed-Effects Linear Regression Analysis of Child Opportunity Index and Life Expectancy at Age 1 Year **eFigure 1.** Flowchart of US Census Tracts Included in the Study **eFigure 2.** Simple Linear Regression Analysis of Indicators of the Child Opportunity Index and Life Expectancy at Birth
