# Supplementary material for: Association of Neighborhood Conditions and Resources for Children With Life Expectancy at Birth in the US
Source: JAMA Netw Open. 2022 Oct 14;5(10):e2235912. doi: 10.1001/jamanetworkopen.2022.35912 (PMC9568807; doi:10.1001/jamanetworkopen.2022.35912)
Supplement: Supplement. — eTable 1. Indicators of the Child Opportunity Index eTable 2. Simple Linear Regression Analysis of Child Opportunity Index and Life Expectancy at Birth eTable 3. Mixed-Effects Linear Regression Analysis of Child Opportunity Index and Life Expectancy at Age 1 Year eFigure 1. Flowchart of US Census Tracts Included in the Study eFigure 2. Simple Linear Regression Analysis of Indicators of the Child Opportunity Index and Life Expectancy at Birth [file jamanetwopen-e2235912-s001.pdf]

Supplementary Online Content

Shanahan KH, Subramanian SV, Burdick KJ, Monuteaux MC, Lee LK, Fleegler EW. Association of neighborhood conditions and resources for children with life expectancy at birth in the US. *JAMA Netw Open*. 2022;5(10):e2235912. doi:10.1001/jamanetworkopen.2022.35912

eTable 1. Indicators of the Child Opportunity Index

eTable 2. Simple Linear Regression Analysis of Child Opportunity Index and Life Expectancy at Birth

eTable 3. Mixed-Effects Linear Regression Analysis of Child Opportunity Index and Life Expectancy at Age 1 Year

eFigure 1. Flowchart of US Census Tracts Included in the Study

eFigure 2. Simple Linear Regression Analysis of Indicators of the Child Opportunity Index and Life Expectancy at Birth

This supplementary material has been provided by the authors to give readers additional information about their work.

© 2022 Shanahan KH et al. *JAMA Network Open*.

eTable 1. Indicators of the Child Opportunity Index

Domain	Indicator
**Education**	*Early childhood education*
	Early childhood education centers<br>High-quality early childhood education centers<br>Early childhood education enrollment
	Elementary education
	Third grade reading proficiency<br>Third grade math proficiency
	Secondary and postsecondary education
	High school graduate rate<br>Advanced Placement course enrollment<br>College enrollment in nearby institutions
	Educational and social resources
	School poverty<br>Teacher experience<br>Adult educational attainment
**Health and Environment**	*Healthy environments*
	Access to healthy food<br>Access to green space<br>Walkability<br>Housing vacancy rate
	Toxic exposures
	Hazardous waste dump sites<br>Industrial pollutants in air, water, or soil<br>Airborne microparticles<br>Ozone concentration<br>Extreme heat exposure
	Health resources
	Health insurance coverage
**Social and Economic**	*Economic Opportunities*
	Employment rate<br>Commute duration
	Economic and social resources
	Poverty rate<br>Public assistance rate<br>Homeownership rate<br>High-skill employment<br>Median household income<br>Single-headed households

© 2022 Shanahan KH et al. *JAMA Network Open*.

eTable 2. Simple Linear Regression Analysis of Child Opportunity Index and Life Expectancy at Birth

Category of Child Opportunity	Model for Composite Score β (95% CI)[a]	Model for Domain-Specific Scores		
		Education β (95% CI)[a]	Health and Environment β (95% CI)[a]	Social and Economic β (95% CI)[a]
Very Low Opportunity	-7.54 (-7.92, -7.17)	-2.69 (-3.10, -2.21)	-2.96 (-3.60, -2.32)	-3.5 (-4.01, -3.01)
Low Opportunity	-4.86 (-5.14, -4.58)	-2.17 (-2.49, -1.86)	-1.89 (-2.32, -1.45)	-1.79 (-2.15, -1.43)
Moderate Opportunity	-3.36 (-3.54, -3.18)	-1.82 (-2.10, -1.59)	-1.33 (-1.57, -1.10)	-1.16 (-1.37, -0.94)
High Opportunity	-1.85 (-1.97, -1.74)	-1.19 (-1.36, -1.02)	-0.79 (-0.93, -0.64)	-0.60 (-0.72, -0.48)
Very High Opportunity[b]	1 [Reference]	1 [Reference]	1 [Reference]	1 [Reference]
R^2 for model	0.41	0.44		

[a]β-coefficient for life expectancy at birth in years (95% confidence interval)
[b]Referent group

© 2022 Shanahan KH et al. *JAMA Network Open*.

eTable 3. Mixed-Effects Linear Regression Analysis of Child Opportunity Index and Life Expectancy at Age 1 Year

Category of Child Opportunity	Model for Composite Score R^2=0.40 β (95% CI)[a]	Model for Domain-Specific Scores R^2=0.43		
		Education β (95% CI)[a]	Health and Environment β (95% CI)[a]	Social and Economic β (95% CI)[a]
Very Low Opportunity	-6.77 (-6.84, -6.70)	-1.93 -(2.02, -1.83)	-2.20 (-2.31, -2.10)	-4.00 (-4.10, -3.90)
Low Opportunity	-4.34 (-4.41, -4.27)	-1.43 (1.51, -1.34)	-1.34 (-1.43, -1.25)	-2.34 (-2.42, -2.25)
Moderate Opportunity	2.90 (-2.97, -2.83)	-1.12 (-1.20, -1.04)	-0.81 (-0.90, -0.74)	-1.53 (-1.61, -1.45)
High Opportunity	-1.54 (-1.61, -1.47)	-0.70 (-0.77, -0.63)	-0.42 (-0.50, -0.35)	-0.83 (-0.90, -0.76)
Very High Opportunity[b]	1 [Reference]	1 [Reference]	1 [Reference]	1 [Reference]

[a]β-coefficient for life expectancy at birth in years (95% confidence interval)
[b]Referent group

© 2022 Shanahan KH et al. *JAMA Network Open*.

eFigure 1. Flowchart of US Census Tracts Included in the Study

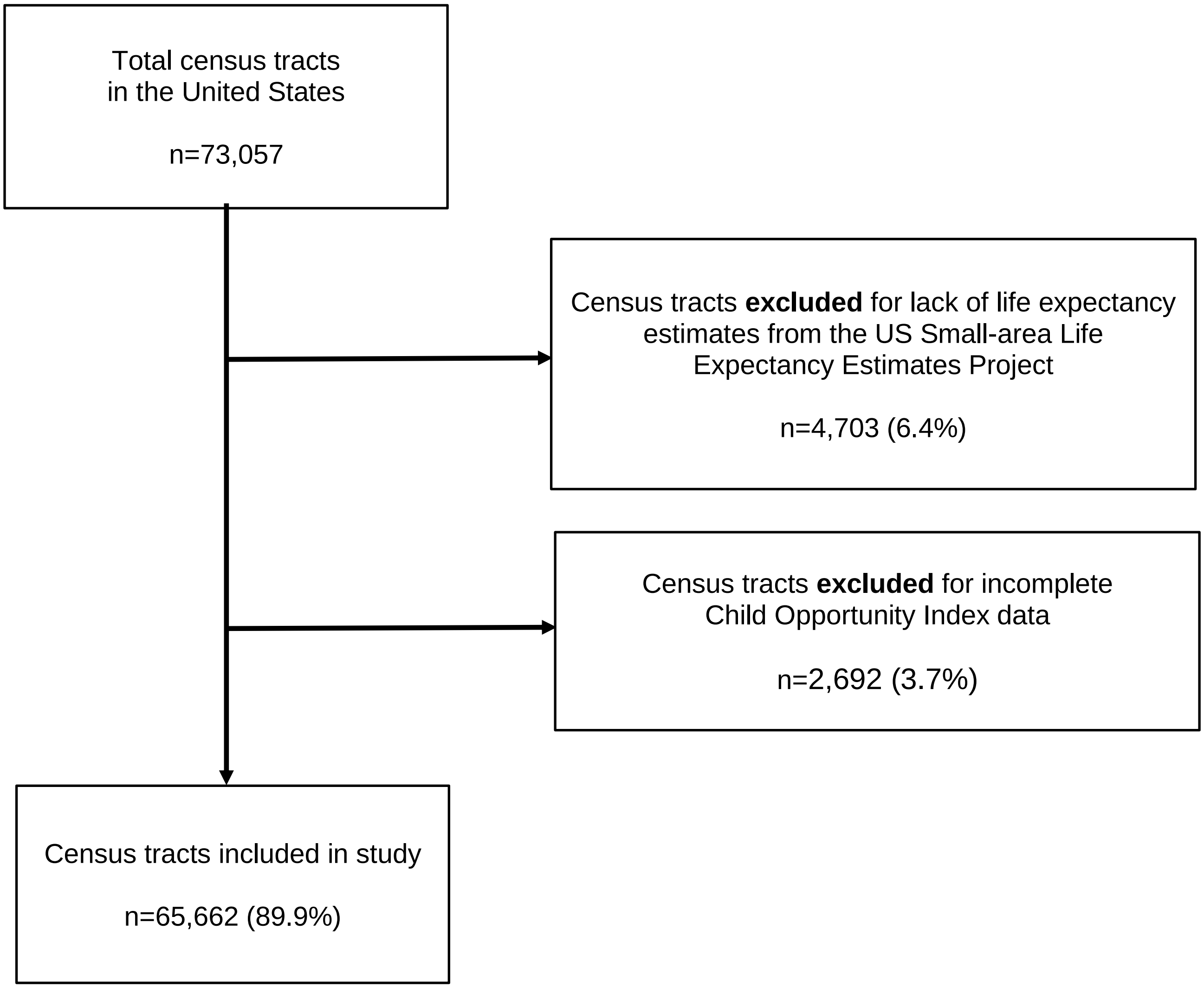

After exclusion for insufficient or absent data from the two sources, 89.9% of all census tracts were included.

© 2022 Shanahan KH et al. *JAMA Network Open*.

eFigure 2. Simple Linear Regression Analysis of Indicators of the Child Opportunity Index and Life Expectancy at Birth

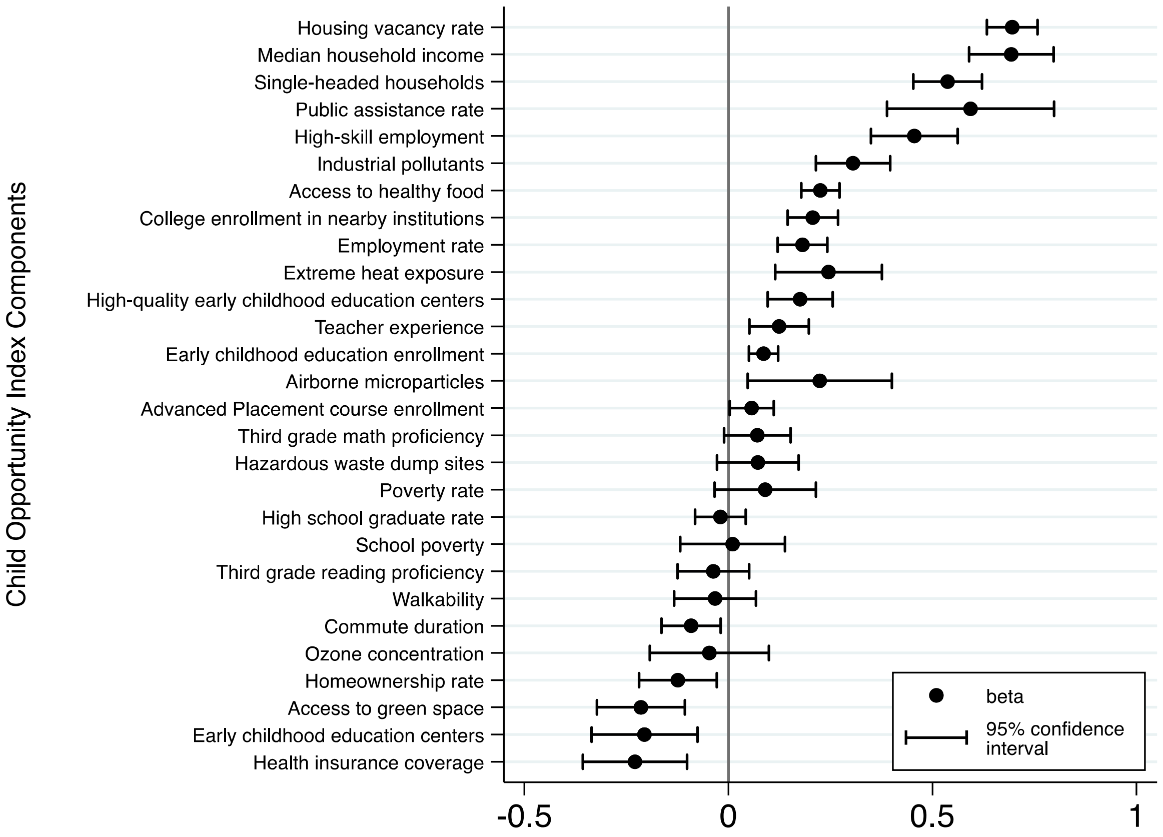

R^2 for the model is 0.54

β-coefficient for life expectancy at birth in years per unit change of indicator (95% confidence interval)

For all indicators, higher scores reflect more favorable conditions and resources for children

© 2022 Shanahan KH et al. *JAMA Network Open*.
